# Coronary Artery Calcium Score as a Predictor of Anthracycline-Induced Cardiotoxicity: The ANTEC Study

**DOI:** 10.3390/ph18081102

**Published:** 2025-07-25

**Authors:** Anna Borowiec, Patrycja Ozdowska, Magdalena Rosinska, Agnieszka Maria Zebrowska, Sławomir Jasek, Beata Kotowicz, Joanna Waniewska, Hanna Kosela-Paterczyk, Elzbieta Lampka, Katarzyna Pogoda, Zbigniew Nowecki, Jan Walewski

**Affiliations:** 1 Non-Commercial Clinical Research Outpatient Clinic, Maria Sklodowska-Curie National Research Institute of Oncology, 02-781 Warsaw, Poland; 2 Department of Cancer & Cardio-Oncology Diagnostics, Maria Sklodowska-Curie National Research Institute of Oncology, 02-781 Warsaw, Poland; 3 Digital Medicine Center, Maria Sklodowska-Curie National Research Institute of Oncology, 02-781 Warsaw, Poland; 4 Unit for Screening Studies in Inherited Cardiovascular Diseases, The Cardinal Stefan Wyszynski National Institute of Cardiology, 04-628 Warsaw, Poland; 5 Cancer Biomarker and Cytokines Laboratory Unit, Maria Sklodowska-Curie National Research Institute of Oncology, 02-781 Warsaw, Poland; 6 Department of Radiology I, Maria Sklodowska-Curie National Research Institute of Oncology, 02-781 Warsaw, Poland; 7 Department of Soft Tissue/Bone Sarcoma and Melanoma, Maria Sklodowska-Curie National Research Institute of Oncology, 02-781 Warsaw, Poland; 8 Department of Lymphoid Malignancies, Maria Sklodowska-Curie National Research Institute of Oncology, 02-781 Warsaw, Poland; 9 Department of Brest Cancer and Reconstructive Surgery, Maria Sklodowska-Curie National Research Institute of Oncology, 02-781 Warsaw, Poland

**Keywords:** cardiotoxicity, atherosclerosis, computed tomography, anthracycline, computed tomographic angiography, coronary artery calcium score, cancer therapy-related cardiovascular toxicity

## Abstract

**Background:** Many risk factors for cancer therapy-related cardiovascular toxicity overlap with risk factors for atherosclerosis. According to the ESC 2022 Cardio-Oncology Guidelines, coronary computed tomography angiography and coronary artery calcium score are not recommended as part of routine risk assessment prior to oncological treatment. The aim of this study was to prospectively assess the influence of coronary artery calcium score (CAC score) on cancer therapy-related cardiac dysfunction in patients with moderate and high risk of cardiovascular toxicity, qualified for anthracycline treatment. **Methods:** In all patients, risk factors were collected, laboratory tests, echocardiography with global longitudinal strain (GLS) assessment and coronary artery tomography with coronary artery calcium score were performed. A total of 80 patients were included in the study, of which 77 (96.25%) were followed for an average of 11.5 months. The mean age at baseline was 60.5 years and 72 (93.51%) were women. **Results:** During observation, five patients (6.49%) died, including two due to heart failure and three due to cancer progression. The majority of patients (59, 76.6%) had breast cancer, 11 (14.3%) were diagnosed with sarcoma and seven (9.1%) with lymphoma. According to the HFA-ICOS risk score, 40 patients (51.9%) were classified as moderate risk (MR), and 37 patients (48.1%) as high risk (HR) for cancer therapy-related cardiovascular toxicity. A CAC score greater than 100 was calculated in 17 (22.1%) patients and greater than 400 in three (3.9%) patients. The CAC score above zero was more common in older patients and in patients classified as high risk (*p* < 0.001). There was also a significant association between CAC score and hypertension, hyperlipidemia, chronic kidney disease, and the level of NT-proBNP. During 12-month follow-up, mild CTRCD occurred in 38 (49.4%) patients, moderate CTRCD was diagnosed in seven (9.1%), and severe in three (3.9%) patients. In the univariable analysis, CTRCD was more common in the high-risk group (*p* = 0.005) and in patients with a CAC score greater than zero (*p* = 0.036). In multivariable analysis, the incidence of CTRCD remains higher in the CAC score > 0 group, even after adjusting for age, hypertension, and hyperlipidemia. In this study group, the CTRCD rates increased with the HFA-ICOS risk score. **Conclusions:** In moderate and high-risk patients, a coronary artery calcium score greater than zero was identified as a significant risk factor for the development of cancer therapy-related cardiac dysfunction during anthracycline-based treatment. Furthermore, the HFA-ICOS risk score demonstrated good correlation with the incidence of CTRCD in this study, supporting its validity as a predictive tool in patients receiving anthracycline therapy.

## 1. Introduction

Oncological treatment, including chemotherapy and radiotherapy, accelerates inflammatory and degenerative processes, contributing to the development of atherosclerosis and cardiovascular complications [1,2,3,4,5]. Patients with cancer who also present with cardiovascular risk factors or pre-existing cardiovascular disease are at heightened risk of cancer therapy-related cardiac dysfunction (CTRCD) [3]. Many of the risk factors for cancer therapy-related cardiovascular toxicity (CTRCVT) overlap with those for atherosclerosis and are accounted for in the Heart Failure Association–International Cardio-Oncology Society (HFA-ICOS) risk tool, currently recommended for pre-treatment cardiovascular risk assessment [3].

Coronary computed tomography angiography (CCTA) is an established non-invasive tool to assess coronary artery disease in asymptomatic individuals in the general population by evaluating the coronary artery calcium (CAC) score and overall atherosclerotic plaque burden [6]. Despite its utility, CCTA and CAC scoring are not currently recommended for routine cardiovascular risk stratification prior to cancer therapy [3]. Nonetheless, CCTA remains the only non-invasive modality capable of directly visualizing coronary arteries and quantifying atherosclerosis. Furthermore, CAC scoring can often be derived from routine chest CT scans, which are frequently performed before initiating cancer therapy.

Recent research suggests a potential cardioprotective role of statins during oncological treatment [7,8,9,10,11,12,13,14,15]. Statins are known to reduce cholesterol levels, inhibit the progression of atherosclerosis, and reduce the risk of atherosclerosis-related complications [16]. The 2022 European Society of Cardiology Cardio-Oncology Guidelines recommend considering statins for the primary prevention of cardiac dysfunction in patients at high or very high risk of cardiovascular toxicity [3]. However, current evidence regarding their effectiveness in preventing chemotherapy-induced cardiotoxicity remains mixed, with some studies reporting benefit [7,14,17], and others showing no significant effect [18,19].

There is a lack of studies evaluating the role of pre-existing, asymptomatic atherosclerotic lesions in contributing to myocardial damage during chemotherapy. This represents a critical gap in cardio-oncology research, especially considering that cancer patients often present with overlapping cardiovascular risk factors. Understanding whether subclinical coronary atherosclerosis predisposes patients to greater myocardial vulnerability during cancer treatment could help refine risk stratification and improve preventive strategies, including more targeted use of cardioprotective agents such as statins.

Therefore, the aim of the ANTEC (Atherosclerosis iN chemoTherapy-rElated Cardiotoxicity) study was to evaluate the prognostic value of CAC score prior to anthracycline chemotherapy in predicting the development of cancer therapy-related cardiac dysfunction. This study is novel in integrating both cardiac functional assessment by echocardiography and subclinical coronary atherosclerosis evaluation by CCTA in a prospective oncology cohort.

We hypothesize that a higher CAC score, indicating the presence of pre-existing asymptomatic coronary atherosclerosis, is associated with an increased risk of developing cardiac dysfunction during anthracycline-based chemotherapy.

## 2. Results

### 2.1. Patients’ Characteristics

**Figure 1:**
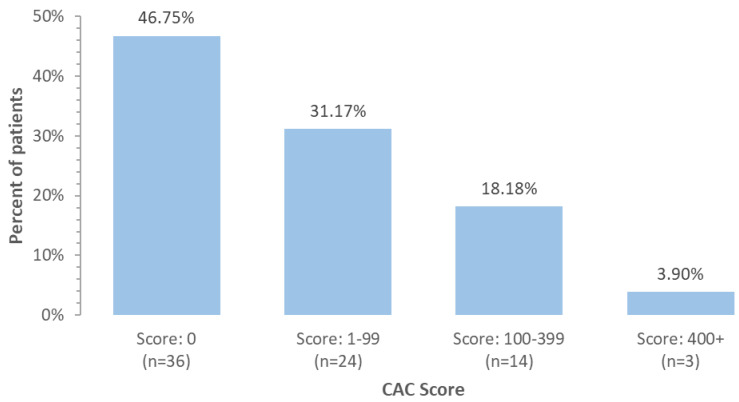
Distribution of coronary artery calcium (CAC) score in cancer patients. Abbreviations: CAC—Coronary Artery Calcium.

**Figure 2:**
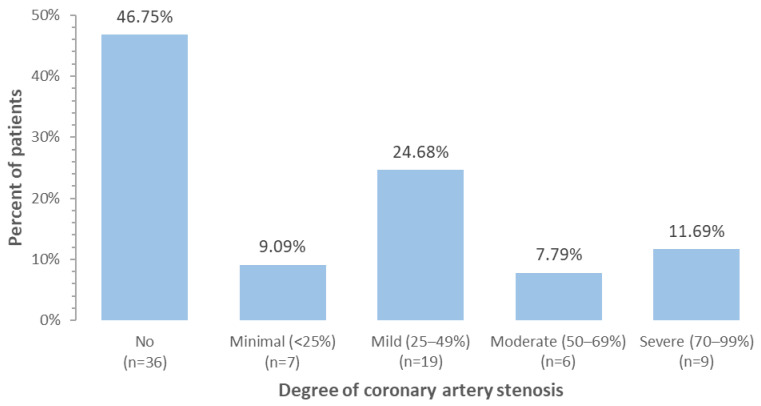
Distribution of coronary artery stenosis in cancer patients.

**Figure 3:**
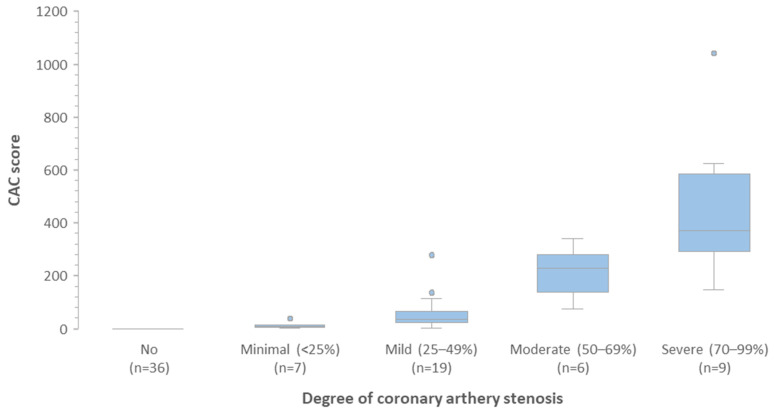
Correlation between coronary artery stenosis and CAC score. Abbreviations: CAC—Coronary Artery Calcium.

According to the protocol, 80 patients were included in the study, of which 77 (96.25%) were followed for 12 months or until the patient died, on average for 11.5 months. The mean age at baseline was 60.5 years and 72 (93.51%) were women. During observation, five patients (6.49%) died, including two due to heart failure and three due to cancer progression. The majority of patients (59, 76.6%) had breast cancer, 11 (14.3%) were diagnosed with sarcoma, and seven (9.1%) with lymphoma. There were 40 (51.9%) patients classified as moderate (MR) and 37 (48.1%) as high risk (HR) of cancer therapy-related cardiovascular toxicity according to current ESC cardio-oncology guidelines. CAC score greater than zero was calculated in 41 (53.2%) patients, greater than 100 in 17 (22.1%) patients and greater than 400 in three (3.9%) patients, Figure 1. About 53.3% of patients had at least minimal coronary artery disease in CCTA, Figure 2. The correlation between CAC score and coronary artery stenosis is presented in Figure 3, showing a high agreement between these two measurements.

At baseline, the mean left ventricle ejection fraction (LVEF) in the entire group was 61.6%, and 5.2% of the patients had an LVEF below 55%. In this study group, the mean anthracycline equivalence dose was 252.29 mg/m^2^, with a cumulative dose range of 60–625 mg/m^2^. No study participant was treated with liposomal doxorubicin or dexrazoxane. The baseline characteristics of the patients are shown in Table 1.

### 2.2. Associations Between Atherosclerosis and Clinical Parameters

The CAC score above zero was more common in older patients and in patients classified as high risk (*p* < 0.001). There was also a significant association between CAC score and hypertension, hyperlipidemia, chronic kidney disease, and NT-proBNP level, Table 2.

### 2.3. Incidence of CTRCD

In general, CTRCD was diagnosed in 48 (62.4%) patients. Mild CTRCD occurs in 38 (49.4%, 95% CI 38.2–60.5%) patients, moderate CTRCD was diagnosed in seven (9.1%, 95% CI 4.3–18.0%), and severe in three (3.9%, 95% CI 1.2–11.6%) patients. Among patients with a CAC score above 100, at least mild CTRCD was diagnosed in 14 of 17 patients (82.4%, 95% CI 56.8–94.3%).

### 2.4. Univariable and Multivariable Analysis

In the univariable analysis, CTRCD was more common in the high-risk group (RR 4.0, 95% CI 1.5–10.9, *p* = 0.005) and in patients with CAC score > 0 (RR 1.5, 95% CI 1.0–2.1, *p* = 0.036), Table 3.

In multivariable analysis, the incidence of CTRCD remains higher in the CAC score > 0 group, after adjusting for other risk factors, Table 4.

### 2.5. Validation of the HFA-ICOS Risk Score

In this study group, CTRCD rates increased with the HFA-ICOS risk score. In the HR group, 29 patients developed CTRCD during follow-up and it occurred more frequently than in the MR group (60.4% vs. 39.6%; *p* = 0.005). Similar results were found for moderate/severe CTRCD. In the HR group, eight patients were diagnosed with moderate or severe CTRCD and in the MR group only two patients (80% vs. 20%; *p* = 0.03).

### 2.6. Deaths

Among the five patients who died, all were women 50 years or older and were classified as high risk for CTR-CVT. Fatal outcomes were observed more frequently in patients with sarcoma than in patients with lymphoma or breast cancer (*p* = 0.01). Furthermore, it was associated with a higher CAC score (*p* = 0.003), lower LVEF (*p* < 0.001), lower GLS (*p* < 0.001), and a higher baseline concentration of NT-proBNP (*p* = 0.043).

## 3. Discussion

The principal finding of this study is that, in patients at moderate and high baseline risk, a CAC score greater than zero significantly increased the risk of developing cancer therapy-related cardiac dysfunction during anthracycline-based treatment. Furthermore, individuals classified as high risk according to the HFA-ICOS baseline cardiovascular toxicity risk stratification tool were more likely to develop CTRCD than those in the moderate-risk category.

### 3.1. Incidence of Atherosclerosis

Coronary artery disease was common in the study population, with 53.3% of patients exhibiting at least minimal coronary artery calcification. This incidence is notably higher than reported in several other studies of oncology patients. For instance, Brann et al. found a similar or less severe CAC burden among patients referred to a cardio-oncology clinic compared to the general population [20]. In a study of breast cancer patients, approximately 24% had a CAC score above zero, and a third of those with elevated CAC scores had no other cardiovascular risk factors [21]. Similarly, Shen et al. reported that 19.4% of patients with diffuse large B-cell lymphoma planned for anthracycline therapy had a CAC score greater than 100 [22]. These disparities may be attributable to our study’s exclusive inclusion of patients at moderate or high risk, among whom traditional cardiovascular risk factors are more prevalent.

### 3.2. Impact of Atherosclerosis on Cardiovascular Complications

Numerous studies have demonstrated the predictive value of CAC scores for acute coronary events [23] and other cardiovascular outcomes in cancer patients [4,5,24,25]. High CAC scores have also been associated with increased mortality. For example, in a large multicenter cohort of 66,636 asymptomatic adults without known cardiovascular disease, elevated CAC independently predicted cardiovascular mortality, even after adjusting for conventional risk factors [26]. Our findings align with this evidence: all five patients who died during the study were classified as high risk for CTR-CVT and exhibited significantly higher CAC scores compared to survivors.

### 3.3. Impact of Atherosclerosis on CTRCD

Prospective data on the relationship between CAC scores and CTRCD are limited, and findings from retrospective studies have been inconsistent. For example, Hooks et al. reported that coronary artery calcification was not an independent predictor of a composite endpoint including myocardial infarction, new-onset heart failure, heart failure hospitalization, and cardiac death. Although calcification predicted coronary events, it did not significantly correlate with heart failure outcomes [2]. In contrast, Shen et al. demonstrated that artificial intelligence-derived CAC measurements from non-gated chest CTs could predict CTRCD in patients with diffuse large B-cell lymphoma receiving anthracyclines [22]. In their study, 24.66% developed CTRCD, a rate higher than in our cohort (13%), possibly due to a longer follow-up period (69 months) and higher cumulative anthracycline doses. Their finding that both mild (1–100) and significant (>100) CAC scores were predictive of CTRCD is consistent with our results.

### 3.4. Validation of the HFA-ICOS Risk Tool

The HFA-ICOS Risk Tool, developed by the European Society of Cardiology and the International Cardio-Oncology Society, stratifies patients based on their baseline risk of cardiotoxicity prior to cancer therapy [3]. Our data demonstrate a strong association between higher HFA-ICOS scores and the incidence of CTRCD among anthracycline-treated patients. Approximately 13% of our patients developed moderate to severe CTRCD. Battisti et al., in a retrospective study of HER2-positive breast cancer patients, reported a lower incidence (5.9%) of LVEF decline, although their cohort included a substantial proportion (43.1%) of low-risk patients [27]. Suntheralingam et al. observed a graded increase in CTRCD across HFA-ICOS risk categories in a trastuzumab-treated cohort: 15.5% in low-risk, 26.9% in moderate-risk, 37.0% in high-risk, and 40.0% in very high-risk patients [28]. Although these studies focused on HER2-positive patients, our results are in line with findings from the CARDIOTOX registry, which showed that approximately 67% of anthracycline-treated patients developed CTRCD and that rates of CTRCD and all-cause mortality increased with the HFA-ICOS score [29]. Similarly, in our study, 62.4% of moderate- and high-risk patients developed CTRCD, with risk positively correlated to HFA-ICOS classification. In contrast, a study by Guerra et al. reported that the HFA-ICOS score had limited predictive value for assessing the risk of CTRCD in breast cancer patients receiving anthracycline and trastuzumab therapy at a national reference center [30].

### 3.5. Strengths and Limitations

This study has several limitations. It was conducted at a single cancer center with a relatively small cohort, potentially limiting generalizability and statistical power. The predominance of female participants, mainly breast cancer patients, may also influence the applicability of findings to broader oncology populations.

Despite these limitations, this study possesses important strengths. It is one of the first prospective investigations to assess the prognostic utility of CAC in patients at moderate and high risk undergoing anthracycline therapy. All participants underwent both coronary computed tomography angiography and echocardiography under standardized protocols, interpreted by the same blinded radiologist and sonographer, thereby minimizing interobserver variability. Moreover, CAC findings were corroborated by CCTA data, strengthening the validity of the observations.

Importantly, our study identifies a CAC score greater than zero as a significant predictor of CTRCD, a distinction that conventional cardiovascular risk factors failed to make. These findings suggest that CAC scoring may offer superior predictive value for CTRCD and should be considered for inclusion in pre-chemotherapy cardiovascular risk assessments.

## 4. Materials and Methods

### 4.1. Study Structure

**Figure 4:**
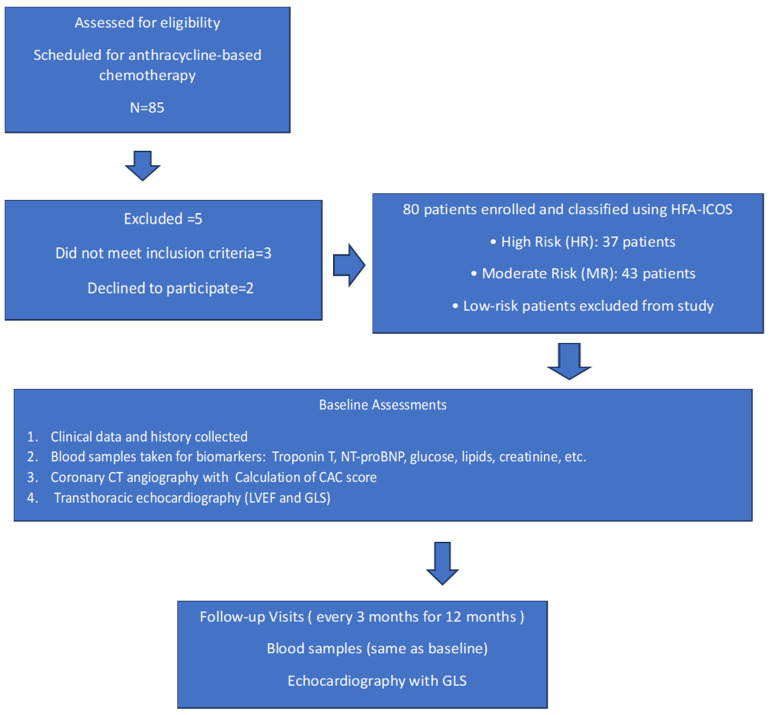
Flowchart for the ANTEC Study.

The ANTEC study is a single center prospective observational study that evaluates the impact of atherosclerosis in the coronary arteries and the CAC score determined by computed tomography on the risk of CTRCD related to anthracycline-based chemotherapy. The study was carried out according to the principles of the Declaration of Helsinki and the guidelines of the International Conference on Harmonization Good Clinical Practice. The conduct of the study was approved by an Independent Ethics Committee at the Maria Sklodowska-Curie National Research Institute of Oncology in Warsaw (date of approval: 28 October 2021, approval no. 80/2021) and all participants provided written informed consent before study entry. The registration identifier on clinicaltrials.gov is NCT05118178. The study included 80 cancer patients classified as moderate (MR) or high risk (HR) of cancer therapy-related cardiovascular toxicity according to the Heart Failure Association–International Cardio-Oncology Society baseline cardiovascular toxicity risk stratification model [3], diagnosed and qualified for systemic treatment with anthracycline chemotherapy at the Maria Sklodowska-Curie National Research Institute of Oncology in Warsaw. The study protocol with inclusion and exclusion criteria, schedule visits, and procedures was previously published [31]. All participants received doxorubicin as the anthracycline agent. Low-risk patients were excluded from the study. None of the patients had previously received any type of chemotherapy or radiotherapy. None of the patients had been diagnosed with coronary artery disease or heart failure. CTRCD was classified as mild, moderate, severe, or very severe according to the ESC 2022 cardio-oncology guidelines [3]. Flowchart for the ANTEC Study is presented in Figure 4.

### 4.2. Patients Classification, CCTA and Echocardiography

The study investigators collected clinical variables based on medical documentation provided by the patient and medical anamnesis. In all patients, coronary computed tomography angiography (CCTA) was performed once at the beginning of the study. The coronary artery calcium (CAC) score was calculated according to the Agatston method. This technique quantifies coronary calcification by identifying areas of increased radiographic density on non-contrast cardiac CT scans. The Agatston score is calculated by multiplying the area of each calcified lesion by a density factor based on the peak attenuation within the lesion. The sum of scores from all coronary arteries represents the total CAC score, which reflects the burden of coronary atherosclerosis [32]. Contrast coronary CTA was performed using a 64-slice multidetector CT scanner (Revolution Evo, GE Healthcare, Waukesha, WI, USA) with prospective ECG triggering. Scans were performed in accordance with the Society of Cardiovascular Computed Tomography guidelines in order to achieve high image quality with the lowest possible patient radiation and contrast dose. The presence, extent and severity of coronary artery stenosis was defined as minimal (<25%), mild (25 to 49%), moderate (50 to 69%), severe (70 to 99%), and occluded (100%) according to guidelines [33].

Transthoracic echocardiography was performed using a Philips EPIQ Elite instrument (Philips Healthcare, Andover, MA, USA). Echocardiographic parameters were evaluated as follows: ventricular diameters, diastolic and systolic functions, and two-dimensional left ventricular peak systolic global longitudinal strain (GLS), which were analyzed using a semi-automated speckle tracking imaging technique from the three standard apical views. The analyses were performed by a board-certified physician who was blinded to all clinical characteristics.

Blood samples were collected and analyzed by the central laboratory (The Maria Sklodowska-Curie National Research Institute of Oncology). Laboratory tests included, among others, creatinine, glucose, complete blood count, glycated hemoglobin, lipid profile, plasma levels of troponin T (TnT), and N-terminal pro-B-type natriuretic peptide (NT-proBNP).

### 4.3. Data Acquisition and Statistical Analysis

#### 4.3.1. Data Acquisition and Quality Control

All measurements were performed according to routine diagnostic standards. Critical measurements (CCTA, echocardiography, biomarkers) were reviewed and coded by a trained member of the study team to ensure comparability of the results. Clinical data was captured by manual entry into electronic case report forms (eCRF) and laboratory data extracted from the Hospital Information System (HIS). The data was validated by the clinical team to eliminate inconsistent, missing, and outlying values.

#### 4.3.2. Sample Size Calculations and Statistical Analysis

The sample size calculation for the ANTEC study was based on its primary objective: to test the hypothesis that the incidence of CTRCD increases twofold—from 30% to 40%—in patients with coronary artery stenosis, which is estimated to be present in 30% to 40% of the study population. Consequently, the current biomarker analysis is exploratory. Descriptive statistics were used to summarize the data. Categorical variables are presented as frequencies and percentages, while continuous variables are reported as means with standard deviations (SD) and 95% confidence intervals (CI), where appropriate. To assess the relationships between examined parameters and established biomarkers, Spearman’s rank correlation coefficients were calculated at baseline and during treatment (using data from Visits 3 and 6). Additionally, repeated measures correlation coefficients were employed to account for within-subject correlations over time. For evaluating biomarkers as potential predictors of CTRCD and biomarker levels measured at Visit 1 (prior to treatment initiation) were analyzed. Furthermore, multivariable random-effects logistic regression models were constructed to examine the association between biomarker levels measured concurrently with CTRCD status and the presence of CTRCD (binary outcome: yes/no). Variables included in the multivariate models were selected based on clinical relevance and prior literature supporting their association with CTRCD. Data collected after the diagnosis of CTRCD were excluded from analyses to minimize confounding effects from toxicity management. Fractional polynomial regression models were applied to determine whether non-linear transformations of predictor variables would improve model fit; however, no significant improvement was observed compared to linear models. All statistical analyses were conducted using Stata/SE version 17.0 (StataCorp LLC, College Station, TX, USA). Statistical significance was set at a two-sided α level of 0.05.

## 5. Conclusions

In patients at moderate and high cardiovascular risk, a coronary artery calcium score greater than zero was identified as a significant risk factor for the development of cancer therapy-related cardiac dysfunction during anthracycline-based treatment. Furthermore, the HFA-ICOS risk score demonstrated good correlation with the incidence of CTRCD in this study, supporting its validity as a predictive tool in patients receiving anthracycline therapy. Incorporating routine CAC score assessment from pre-treatment CT scans could provide valuable insights into patients’ cardiovascular risk profiles and support the timely initiation of cardioprotective therapy. Since the CAC score can be easily obtained from standard pre-chemotherapy CT imaging, it represents a quick and accessible risk marker that could serve as a deciding factor in initiating lipid-lowering treatment in selected patients as part of CTRCD prevention. We hope that the results of this study will support the inclusion of the CAC score in standard risk assessment prior to treatment initiation and facilitate a more accurate evaluation of patients before oncologic therapy, ultimately helping to reduce the risk of cardiotoxicity in the future.

## Figures and Tables

**Table 1:** Baseline characteristics of the study group.

Characteristics of the Patients, n = 77		Number of Patients, %
Age group (years)	<65	41 (53.2%)
	≥65	36 (46.8%)
Cancer	breast	59 (76.6%)
	lymphoma	7 (9.1%)
	sarcoma	11 (14.3%)
Risk group	High Risk	37 (48.1%)
	Moderate Risk	40 (51.9%)
BMI	<25	24 (31.2%)
	25–30	28 (36.4%)
	˃30	25 (32.5%)
Coexisting Conditions *	Hypertension	60 (77.9%)
	Hyperlipidemia	58 (75.3%)
	Diabetes	11 (14.3%)
	Chronic kidney disease	12 (15.6%)
	Ever smoke	37 (48.1%)
NYHA scale	1	54 (70.1%)
	2	23 (29.9%)
ECOG score	0	55 (71.4%)
	1	21 (27.3%)
	2	1 (1.3%)
Medications *	Beta-blockers	33 (42.9%)
	ACE-I	34 (44.2%)
	Statins	23 (29.9%)
Coronary artery stenosis	No	36 (46.8%)
	Minimal (<25%)	7 (9.1%)
	Mild (25–49%)	19 (24.7%)
	Moderate (50–69%)	6 (7.8%)
	Severe (70–99%)	9 (11.7%)
Coronary artery calcium (CAC) score	0	36 (46.8%)
	1–99	24 (31.2%)
	100–399	14 (18.2%)
	400+	3 (3.9%)
Anthracycline dose (mg/m^2^)	<250	62 (80.5%)
	≥250	15 (19.5%)
Troponin T (ng/L)	≤14	71 (92.2%)
	>14	6 (7.8%)
NT-proBNP (pg/mL)	<125	48 (62.3%)
	≥125	29 (37.7%)
Left ventricle ejection fraction, LVEF (%)	≥55%	73 (94.8%)
	50–54%	4 (5.2%)

Abbreviations: ACE-I—Angiotensin-Converting Enzyme Inhibitor; BMI—Body Mass Index; CAC—Coronary Artery Calcium; ECOG—Eastern Cooperative Oncology Group; LVEF—Left Ventricular Ejection Fraction; NT-proBNP—N-terminal pro-B-type Natriuretic Peptide; NYHA—New York Heart Association. * Categories may overlap.

**Table 2:** Coronary artery disease (CAD) in cancer patients (n = 77).

Factor		CAC Score = 0 n = 36	CAC Score > 0 n = 41	*p*-Values
Age group	<65	28 (77.8%)	13 (31.7%)	<0.001
	65	8 (22.2%)	28 (68.3%)	
Cancer	breast	26 (72.2%)	33 (80.5%)	0.389
	lymphoma	5 (13.9%)	2 (4.9%)	
	sarcoma	5 (13.9%)	6 (14.6%)	
Anthracycline dose (mg/m^2^)	<250	28 (77.8%)	34 (82.9%)	0.569
	≥250	8 (22.2%)	7 (17.1%)	
Risk group	HR	6 (16.7%)	31 (75.6%)	<0.001
	MR	30 (83.3%)	10 (24.4%)	
BMI	<25	12 (33.3%)	12 (29.3%)	0.712
	25–30	14 (38.9%)	14 (34.1%)	
	≥30	10 (27.8%)	15 (36.6%)	
Hypertension	No	12 (33.3%)	5 (12.2%)	0.026
	Yes	24 (66.7%)	36 (87.8%)	
Hyperlipidemia	No	14 (38.9%)	5 (12.2%)	0.007
	Yes	22 (61.1%)	36 (87.8%)	
Ever smoke	No	20 (55.6%)	20 (48.8%)	0.553
	Yes	16 (44.4%)	21 (51.2%)	
Diabetes	No	33 (91.7%)	33 (80.5%)	0.162
	Yes	3 (8.3%)	8 (19.5%)	
Chronic kidney disease	No	35 (97.2%)	30 (73.2%)	0.004
	Yes	1 (2.8%)	11 (26.8%)	
NT-proBNP	<125	28 (77.8%)	20 (48.8%)	0.009
	≥125	8 (22.2%)	21 (51.2%)	
GLS	<16%	2 (5.6%)	7 (17.1%)	0.117
	≥16%	34 (94.4%)	34 (82.9%)	
LVEF	≥55%	35 (97.2%)	38 (92.7%)	0.370
	50–54%	1 (2.8%)	3 (7.3%)	

Abbreviations: CAC—Coronary Artery Calcium; HR—High Risk; MR—Moderate Risk; BMI—Body Mass Index; NT-proBNP—N-terminal pro-B-type Natriuretic Peptide; GLS—Global Longitudinal Strain; LVEF—Left Ventricular Ejection Fraction.

**Table 3:** Incidence of CTRCD in cancer patients (n = 77).

		n CTRCD/n Total	CTRCD Incidence, 95% CI	Univariable Risk Ratio for CTRCD, 95% CI	*p*-Value
Age group	<65	23/41	56.1% (40.6–70.5%)	-	0.228
	≥65	25/36	69.4% (52.5–82.4%)	1.2 (0.9–1.8)	
Cancer	Breast	37/59	62.7% (49.6–74.2%)	-	0.440
	Lymphoma	3/7	42.9% (14.1–77.4%)	0.7 (0.3–1.6)	
	Sarcoma	8/11	72.7% (40.9–91.1%)	1.2 (0.8–1.8)	
Risk group	HR	29/37	78.4% (62.1–88.9%)	4.0 (1.5–10.9)	0.005
	MR	19/40	47.5% (32.5–63.0%)	-	
BMI	<30	31/54	57.4% (43.8–70.0%)	-	0.171
	≥30	17/23	73.9% (52.4–87.9%)	1.3 (0.9–1.8)	
Hypertension	No	8/17	47.1% (25.2–70.1%)	-	0.141
	Yes	40/60	66.7% (53.7–77.5%)	1.4 (0.8–2.4)	
Hyperlipidemia	No	11/19	57.9% (35.3–77.6%)	-	0.645
	Yes	37/58	63.8% (50.6–75.2%)	1.1 (0.7–1.7)	
Ever smoke	No	29/40	72.5% (56.6–84.2%)	-	0.056
	Yes	19/37	51.4% (35.4–67.0%)	0.7 (0.5–1.0)	
Diabetes	No	41/66	62.1% (49.7–73.1%)	-	0.924
	Yes	7/11	63.6% (33.4–85.9%)	1.0 (0.6–1.7)	
Chronic kidney disease	No	38/65	58.5% (46.0–69.9%)	-	0.102
	Yes	10/12	83.3% (51.7–95.9%)	1.4 (1.0–2.0)	
NYHA scale	1	32/54	59.3% (45.6–71.6%)	-	0.393
	2	16/23	69.6% (48.1–84.9%)	1.2 (0.8–1.7)	
ECOG score	0	32/55	58.2% (44.7–70.6%)	-	0.417
	1	15/21	71.4% (48.9–86.7%)	1.2 (0.9–1.7)	
	2	1/1	100.0%	-	
Beta-blockers	No	27/44	61.4% (46.2–74.6%)	-	0.839
	Yes	21/33	63.6% (46.0–78.3%)	1.0 (0.7–1.5)	
ACE-i	No	26/43	60.5% (45.1–74.0%)	-	0.703
	Yes	22/34	64.7% (47.3–78.9%)	1.1 (0.8–1.5)	
Statins	No	32/54	59.3% (45.6–71.6%)	-	0.393
	Yes	16/23	69.6% (48.1–84.9%)	1.2 (0.8–1.7)	
CAC score	0	18/36	50.0% (34.0–66.0%)	-	0.036
	>0	30/41	73.2% (57.5–84.6%)	1.5 (1.0–2.1)	
Anthracycline dose (mg/m^2^)	<200	9/13	69.2% (40.5–88.2%)	-	0.793
	200–400	33/55	60.0% (46.4–72.2%)	0.9 (0.6–1.3)	
	˃400	6/9	66.7% (32.8–89.1%)	1.0 (0.5–1.7)	
Troponin T	<14	44/71	62.0% (50.0–72.6%)	-	0.820
	≥14	4/6	66.7% (26.3–91.8%)	1.1 (0.6–1.9)	
NT-proBNP	<125	27/48	56.2% (41.9–69.7%)	-	0.156
	≥125	21/29	72.4% (53.4–85.7%)	1.3 (0.9–1.8)	
LVEF (%)	≥55	45/73	61.6% (49.9–72.2%)	-	0.591
	50–54	3/4	75.0% (23.1–96.8%)	1.2 (0.7–2.2)	

Abbreviations: CTRCD—Cancer Therapy-Related Cardiac Dysfunction; BMI—Body Mass Index; HR—High Risk; MR—Moderate Risk; NYHA—New York Heart Association Functional Classification; ECOG—Eastern Cooperative Oncology Group Performance Status; ACE-i—Angiotensin-Converting Enzyme Inhibitor; CAC—Coronary Artery Calcium; NT-proBNP—N-terminal pro-B-type Natriuretic Peptide; LVEF—Left Ventricular Ejection Fraction.

**Table 4:** Results of adjusted analysis of the impact of the presence of the coronary artery calcium score on CTRCD (n = 77).

		Multivariable Risk Ratio for CTRCD, 95% CI	*p*-Value
Adjustment for age group, hypertension and hyperlipidemia			
CAC score > 0	No	Reference	
	Yes	1.53 (1.06–2.21)	0.0247
Age group	24–49	Reference	
	50–64	1.55 (0.85–2.83)	0.1496
	65–80	1.2 (0.65–2.22)	0.5524
Hypertension	No	Reference	
	Yes	1.48 (0.83–2.63)	0.1805
Hyperlipidemia	No	Reference	
	Yes	1.03 (0.66–1.61)	0.9094

Abbreviations: CTRCD—Cancer Therapy-Related Cardiac Dysfunction; CAC score—Coronary Artery Calcium score.

## Data Availability

The data presented in this study are available on request from the corresponding author. The data are not publicly available due to ethical reasons.

## Abbreviations

CTRCD	Cancer therapy-related cardiac dysfunction
CTR-CVT	Cancer therapy-related cardiovascular toxicity
HFA-ICOS	Heart Failure Association–International Cardio-Oncology Society
CCTA	Coronary computed tomography angiography
CAC score	Coronary artery calcium score
GLS	Left ventricular peak systolic global longitudinal strain
hs-cTnT	High-sensitivity cardiac troponin T
NT-proBNP	N-terminal pro-B-type natriuretic peptide
eCRF	Electronic case report forms
HIS	Hospital Information System
LVEF	Left ventricle ejection fraction
CVD	Cardiovascular disease
